# A growing link—what is the role of height in cancer risk?

**DOI:** 10.1038/s41416-018-0370-9

**Published:** 2019-02-19

**Authors:** Edward Giovannucci

**Affiliations:** Harvard T.H. Chan School of Public Health, 665 Huntington Ave, Boston, MA 02115 USA

## Abstract

A recent study has examined adult height in relation to cancer risk in a cohort of 23 million Korean adults. Taller stature was associated with higher risk of every cancer studied, except for oesophageal cancer. This association contributes to our understanding of cancer and may help aid in cancer risk prediction.

## Main

The association between taller stature and higher risk of many cancers is remarkably robust. The relationship is fairly linear, and is observed for different ethnicities, for both sexes, and for most cancers. In this issue of the *British Journal of Cancer*, Choi et al. report on a study of adult height in relation to cancer risk in a cohort of 23 million Korean adults.^1^ For every site examined—about two dozen—with the exception of oesophageal cancer, increased adult height was associated with a higher risk of cancer. These findings extend the current published literature, which is derived predominately from white populations in Europe and North America.^2^ The most consistent findings from these published studies are an increased risk of cancers of the nervous system, thyroid, breast, lung, colon, rectum, prostate, ovary, testes, cervix, endometrium and skin, as well as lymphoma, multiple myeloma and leukaemia, with increased height. Choi et al. also observed an association between height and cancers of the oral cavity, urinary bladder and pancreas, liver, and stomach, which have been less consistently associated with height.^2^ The large sample size of the Korean study undoubtedly aided in identifying these associations.

The magnitude of the association (per unit increment in height) is stronger in women than men for most cancer sites, and is stronger in non-smokers compared to smokers. The stronger association between height and cancer in women could in part be explained by the generally lower rates of smoking in women. In addition, the correlation between height and a certain cancer might be potentially masked or diluted when a particularly strong risk factor exists for that cancer.^3^ Accordingly, associations have been less consistent for gastric cancer, determinants for which include *Helicobacter pylori* infections, which might be related to lower socioeconomic status, crowding, and reduced height.

## A complex variable

Although simple to measure, height is a complex variable that is downstream of multiple biological and sociological determinants. When a population experiences increased availability in energy and protein-rich foods, a decrease in physical activity and a reduced incidence in childhood parasitic and diarrhoeal infections, the population’s average height is increased. The role of the childhood environment is observed through the increase in body height during the 20th century, which was concurrent with the increase in the standard of living.^4^ Within populations that are already economically developed, and for which a stable height has been established, the determinants of height can be largely genetic; in this situation, genetic variation in height is associated with cancer risk (as shown by Mendelian randomisation studies).^5^ The main determinant of height across different populations is economic development and nutrition rather than genetics, and notably, the average population height between countries also correlates with cancer rates.^6^ Thus, both genetic and environmental determinants contribute to height and, consequently, to the risk of cancer.

As indicated by Green et al.,^2^ the increase in average adult height in European populations, by about 1 cm per decade throughout the 20th century, would predict a 10–15% increase in cancer incidence above that expected if the height had not changed over this time period. In the Korean study,^1^ the average height of the population increased by about 2 cm per decade in the latter half of the 20th century. In addition to changes in other cancer risk factors (smoking, alcohol, obesity, infections, screening, etc.), this increase in height likely contributed substantially to the rise in overall cancer incidence in Korea over the past 50 years.

## Deciphering the mechanisms

Does the association between stature and cancer incidence provide clues to cancer aetiology? How an increase in height confers a higher risk of cancer is complicated by the fact that the biological determinants of height are multifactorial. Despite this, two general effects have been proposed: in the first (direct) effect, increased height reflects more stem cells that are at risk of acquiring driver mutations during cell division over time; in the second (indirect) effect, a common factor (such as insulin-like growth factor (IGF) 1) directly affects cancer risk as well as increasing height. Nunney^7^ has used modelling to support the importance of the direct effect for most cancers, although the association between height and melanoma risk was stronger than that predicted solely by cell number. Thus, an increase in cell number and cell divisions marked by taller stature could at least partly explain the increased cancer risk.^8^

One of the more attractive hypotheses for an indirect effect relates to IGF1. IGF1 level is clearly one of the major determinants of height, and circulating IGF1 level is associated with an increased risk of a number of cancers.^9^ Yet, Mendelian randomisation studies demonstrate that the genetic component related to height is also associated with cancer risk, and the majority of the variants are not IGF1-related.^5^ These results indicate that IGF1 cannot be the sole factor linking height to cancer risk. Nonetheless, IGF1 levels are among the determinants of organ size, and thus IGF1 could be part of the story, even if the ultimate mediator is greater cell division.

Whether the magnitudes of the associations between height and various cancers are strong enough to be of clinical relevance (e.g., for risk prediction) is unclear. Perhaps more direct measures of organ size could more precisely estimate risk. Notably, autopsy studies have revealed that total body weight (presumably fat-free weight) correlates even more strongly with organ size than does height.^10^ The larger organ sizes in men relative to women, and in larger individuals relative to smaller ones, could partly explain the different incidences in non-sex-related cancers by sex and body size. The differences in size are substantial—many organs (e.g., heart, lung, liver, kidney, thyroid) are approximately twice as large in a moderately large male (1 standard deviation above the mean) compared to a moderately small female (1 standard deviation below the mean). Assuming that organ size proportionally reflects the number of cells at risk, this variation in organ size might be a more clinically meaningful predictor of cancer risk.

## References

[1] Choi, Y.J. et al. Adult height in relation to risk of cancer in a cohort of 22,809,722 Korean adults. *Br. J. Cancer* (2019); 10.1038/s41416-018-0371-8. 10.1038/s41416-018-0371-8 PMC6462046 30778143

[2] Green J (2011). Height and cancer incidence in the Million Women Study: prospective cohort, and meta-analysis of prospective studies of height and total cancer risk. Lancet Oncol..

[3] Albanes D (1998). Height, early energy intake, and cancer. Evidence mounts for the relation of energy intake to adult malignancies. BMJ.

[4] Silventoinen K (2003). Determinants of variation in adult body height. J. Biosoc. Sci..

[5] Zhang, B. et al. Height and breast cancer risk: evidence from prospective studies and mendelian randomization. *J. Natl. Cancer Inst.* **11**, djv219 (2015). 10.1093/jnci/djv219 PMC4643630 26296642

[6] Albanes D, Taylor PR (1990). International differences in body height and weight and their relationship to cancer incidence. Nutr. Cancer.

[7] Nunney L (2018). Size matters: height, cell number and a person’s risk of cancer. Proc. Biol. Sci..

[8] Albanes D, Winick W (1988). Are cell number and cell proliferation risk factors for cancer?. J. Natl. Cancer Inst..

[9] Giovannucci E (2003). Nutrition, insulin, insulin-like growth factors and cancer. Horm. Metab. Res..

[10] Kim Y (2009). Statistical analysis for organ weights in Korean adult autopsies. Korean J. Anat..

